# Effect of intensive multifactorial treatment on vascular progenitor cells in hypertensive patients

**DOI:** 10.1371/journal.pone.0190494

**Published:** 2018-01-05

**Authors:** Charbel Maroun-Eid, Adriana Ortega-Hernández, Javier Modrego, María Abad-Cardiel, José Antonio García-Donaire, Leonardo Reinares, Nieves Martell-Claros, Dulcenombre Gómez-Garre

**Affiliations:** 1 Unit of Hypertension, Área de Prevención Cardiovascular, Hospital Clínico San Carlos, Instituto de Investigación Sanitaria del Hospital Clínico San Carlos (IdISSC), Madrid, Spain; 2 Vascular Biology Research Laboratory, Hospital Clínico San Carlos-IdISSC, Madrid, Spain; 3 Biomedical Research Networking Center in Cardiovascular Diseases (CIBERCV), Madrid, Spain; 4 Unit of Lipids, Área de Prevención Cardiovascular, Hospital Clínico San Carlos-IdISSC, Madrid, Spain; Universita degli Studi di Perugia, ITALY

## Abstract

**Background:**

Most hypertensive patients, despite a proper control of their cardiovascular risk factors, have cardiovascular complications, evidencing the importance of controlling and/or reversing target-organ damage. In this sense, endothelial dysfunction has been associated with the presence of cardiovascular risk factors and related cardiovascular outcomes. Since hypertension often clusters with other risk factors such as dyslipemia, diabetes and obesity, in this study we have investigated the effect of intensive multifactorial treatment on circulating vascular progenitor cell levels on high-risk hypertensive patients.

**Design:**

We included108 hypertensive patients receiving intensive multifactorial pharmacologic treatment and dietary recommendations targeting blood pressure, dyslipemia, hyperglycemia and weight for 12 months. After the treatment period, blood samples were collected and circulating levels of endothelial (CD34+/KDR+, CD34+/VE-cadherin+) and smooth muscle (CD14+/endoglin+) progenitor cells were identified by flow cytometry. Additionally, plasma concentration of vascular endothelial growth factor (VEGF) was determined by ELISA.

**Results:**

Most hypertensive patients (61±12 years, 47% men) showed cardiovascular parameters within normal ranges at baseline. Moreover, body mass index and the majority of the biochemical parameters (systolic and diastolic blood pressure, fasting glucose, total cholesterol, HDL-c, LDL-c, creatinine and hs-CRP) significantly decreased overtime. After 12 months of intensive treatment, CD34+/KDR+ and CD14+/endoglin+ levels did not change, but CD34+/VE-cadherin+ cells increased significantly at month 12 [0.9(0.05–0.14)% vs 0.05(0.02–0.09)% P<0.05]. However, VEGF plasma concentration decreased significantly overtime [89.1(53.9–218.7) vs [66.2(47.5–104.6) pg/mL, P<0.05].

**Conclusions:**

Long-term intensive treatment in hypertensive patients further improves cardiovascular risk and increases circulating EPCs, suggesting that these cells could be a therapeutic target.

## Introduction

In the past, the goal of treating hypertension was merely blood pressure (BP) reduction, and antihypertensive therapy demonstrated to diminish cardiovascular events around 25% [1]. Updated guidelines target reductions in overall cardiovascular risk since hypertension usually occurs in association with other major risk factors. However, optimally treated hypertensive patients still have an around 50% increased risk of any cardiovascular event [2].

The balance between endothelial injury and endothelial recovery is critical to the reduction of cardiovascular events [3]. The injured vessels release circulating endothelial cells (CECs) and endothelial microparticles (EMPs), and their determination have demonstrated to closely reflect the status of activated/damaged endothelium [4].However, endothelial cells have limited capacity for regeneration, contrasting to the traditional concept that the repair of vascular endothelium was achieved by neighboring endothelial proliferation [5]. Since the identification of endothelial progenitor cells (EPCs) by Asahara et al., and their capacity to differentiate into mature cells and restore endothelial integrity and function has been evidenced, there has been a growing interest in their involvement in cardiovascular disease [6, 7]. The balance between endothelial fragmentation into EMPs and endothelial repair by EPCs has been defined as “vascular competence” of each individual [4]. In this sense, in untreated hypercholesterolemic patients, Pirro et al. have reported an increased ratio of EMPs/EPCs, as well as a positive correlation with aortic stiffness, a reliable marker of atherosclerosis [8].

Circulating EPCs are bone marrow derived cells characterized by the expression of both hematopoietic (CD34, CD133) and endothelial [KDR (a vascular endothelial growth factor receptor), VE-cadherin, von Willebrand factor or CD31] surface markers [9] that have demonstrated their capacity to maintain the integrity of the blood vessels by homing into sites of endothelial injury and differentiating into mature endothelium [10, 11]. Increasing evidence suggest that cardiovascular risk factors associated with endothelial dysfunction, affect the amount and properties of the EPCs [12, 13]. For example, chronic smokers have endothelial dysfunction and it has been reported that smoking cessation led to a rapid restoration of EPCs levels [14]. As well, a low number of EPCs were found in subjects with diabetes mellitus type 2 [15]. In addition, in chronic exposure to increased plasma cholesterol levels, the availability of EPCs is reduced [16]. In fact, the number of EPCs is a better indicator of endothelial dysfunction than the Framingham risk score [13]. The depletion in the number of EPCs has been used as a biomarker of the occurrence of a first major cardiovascular event in patients at different risks or even in healthy subjects [17, 18]. Moreover, restoration of EPC number and/or functionality is possible through current therapies for cardiovascular risk factors and other means [19–21], suggesting that they could also be a promising tool for measuring therapeutic efficacy.

In the last years, the participation in vascular diseases of another circulating bone marrow-derived progenitor cell population has been reported [22]. In this sense, cells expressing CD14/endoglin have been identified as circulating smooth muscle progenitor cells (SMPCs) and associated with the formation of intimal lesions in experimental models [23]. Patients with coronary artery disease (CAD) show higher number of SMPCs than patients without CAD [24].

Previously, we have reported that the number of EPCs in treated hypertensive patients was not normalized despite showing cardiovascular parameters within normal ranges [25]. Thus, the aim of the present study was to evaluate the effect of a long multifactorial intensified treatment on the number of different phenotypes of circulating vascular progenitor cells on high cardiovascular risk hypertensive patients in ordering to justify their use as biomarkers of therapeutic efficacy.

## Material and methods

### Study population

Consecutive consenting adult hypertensive patients with moderate, high or very high cardiovascular risk according to 2007 European Society of Hypertension/European Society of Cardiology (ESH/ESC) Guidelines for the management of arterial hypertension [26] who attended the Hypertension Unit of Hospital Clínico San Carlos were included in the study (n = 108). Very high-risk patient were those having an established cardiovascular or renal disease, at high cardiovascular risk when the patient had 3 or more risk factors, organ damage or diabetes mellitus, and moderate cardiovascular risk when the patient has one or two risk factors. Patients with acute myocardial infarction (< 3 months), acute or chronic inflammatory or malignant disease, as well as any other pathology which might interfere with the study results at the investigator’s discretion were excluded from the study.

Once the patient was included in the study, the treatment of all cardiovascular risk factors were reviewed and optimized aiming to achieve the highest therapeutic targets suggested by the guidelines for the management of arterial hypertension: systolic/diastolic BP (SBP/DBP) ≤140/90 mmHg, total cholesterol <175 mg/dL, LDL-cholesterol <100 mg/dL (80 mg/dL if possible), triglycerides <150 mg/dL, HbA1c ≤ 7%, and smoking cessation. For this purpose, all patients were prescribed an angiotensin converting enzyme (ACE) inhibitor or, if such a drug was contraindicated, an angiotensin II receptor antagonist (ARAII) irrespective of the BP level. If a patient had SBP/DBP >140/90 mmHg, diuretics, calcium channel blockers, and beta-blockers were added as needed. The combination of an ACE inhibitor and an ARAII could also be used. Patients were also prescribed a statin irrespective of fasting serum cholesterol concentration. Fibrates were added to statin treatment if the triglyceride concentration was also elevated (>350 mg/dL).If patients showed HbA1c >7%, an oral antidiabetic agent was started, and if high HbA1c value persisted they were added or switched to insulin. When indicated, platelet antiaggregants or oral anticoagulants were also prescribed. All smoking patients were invited to participate in smoking-cessation programs. Simultaneously, patients were recommended non-drug therapy, including weight reduction, moderation in the consumption of alcohol, physical exercise, reduced salt intake and follow a Mediterranean diet type or a Dietary Approaches to Stop Hypertension diet (DASH diet). During the study, patients were asked about their adherence to the recommendations in each visit. Patients were followed up by means of clinic visits one month after their inclusion in the study and every third month. After 12 months of follow up, we proceeded to the final visit in which all determinations of baseline (clinical biochemical variables, and quantification of progenitor cells and VEGF) were repeated.

The protocol of this study complies with the principles of the Helsinki Declaration and has been approved by the Ethics and Clinical Investigation Committee of Hospital Clínico San Carlos. Informed consent was obtained from all subjects.

### Clinical and laboratory measurements

Medical records were carefully reviewed at an interview, and a thorough physical examination was performed. Gender, age, anthropometric measurements (weight, height), SBP/DBP, smoking habit and personal history was recorded. Body mass index (BMI) was calculated.

A sample of fasting venous blood was drawn for routine biochemical measurements and Service of Clinical Biochemistry at the Hospital Clínico San Carlos performed all analysis unless noted otherwise.

### Quantification of circulating vascular progenitor cells

Blood samples were processed within four hours after collection. Peripheral blood cells were analyzed by direct flow cytometry as previously described [25]. Briefly, 100 μl of blood was incubated on ice protected from light with specific antibodies: anti-CD34 phycoerythrin-cyanin 7 (PC7)-conjugated (mouse IgG1, Beckman Coulter), anti-CD3 phycoerythrin-Texas Red-x (ECD)-conjugated (mouse IgG1, Beckman Coulter), anti-KDR phycoerythrin (PE)-conjugated (mouse IgG1, R&D Systems), anti-VE-cadherin(CD144) PE-conjugated (mouse IgG2b, R&D Systems), anti-CD14 PC7-conjugated (mouse IgG1, Beckman Coulter), and anti-endoglin(CD105) PE-conjugated (mouse IgG2b R&D Systems). Appropriate isotype controls were used for each staining procedure. After 30 minutes of incubation, red blood cells were lysed by a commercial lysis solution (BD FACS Lysis solution, Becton Dickinson) for 10 minutes at room temperature and washing in PBS. After two washing steps, cells were resuspended in 300 μl of PBS and acquired on a FC500 flow cytometer (Beckman Coulter). Data are expressed as percentage of positive cells CD34/KDR, CD34/VE-cadherin and CD14/endoglin.

### Measurement of vascular endothelial growth factor (VEGF)

VEGF levels were determined using a commercially available kit (Quantikine, R&D Systems, UK). The concentrations were determined by comparison with a standard curve, following the manufacturer’s instruction.

### Statistical analyses

Qualitative variables were summarized by their frequency distribution as well as quantitative variables by their mean and standard deviation (± SD). The continuous non-normally distributed variables were summarized by the median and interquartile range (IQR: P25-P75). The Kolmogorov-Smirnov test was used to prove Gaussian distribution. In case of qualitative variables, comparison was evaluated by the test of χ2. For continuous normally distribute variables the T-Student test was used to compare two groups. The Mann-Whitney U test was used for continuous not normally distributed variables. The association between continuous variables was tested using the non-parametric Spearman’s correlations coefficient. As circulating vascular progenitor cells were not normally distributed, these data were log-transformed to improve their distribution for statistical testing, with back-transformed results for presentation in figures and tables. A multivariate linear regression analysis was fitted in order to evaluate the variables associated with circulating vascular progenitor cells. Adjustment was with those variables which, in the univariate analyses, showed a level of statistical significance of P<0.05, and/or were considered clinically relevant. Null hypothesis was rejected by a type I error minor than 0.05 (P<0.05). Statistical analyses were performed using the SPSS 17.0 statistical package.

## Results

### Effect of intensive therapy on circulating vascular progenitor cell levels

The clinical, biochemical characteristics of the participants and their treatments at start and after treatment intensification during 12 months are presented in Table 1. Within the clinical history of our hypertensive patients, 67.6% showed also dyslipidemia, 31.5% diabetes mellitus, 30.6% have been suffered or suffer cardiovascular complications (heart disease, advanced retinopathy, peripheral artery disease, stroke and/or chronic kidney disease), and 14.8% were current smokers. By exception BMI, patients presented a good control of their cardiovascular risk factors but the multifactorial intensive treatment for 12 months improved them even more (Table 1).

**Table 1:** Clinical-biochemical parameters and drug treatment of hypertensive patients at start (baseline) and after treatment intensification during 12 months.

	Hypertensive patients (n = 108)	
	Baseline	12 months
Age (years)	61.0 ± 12.0	
Gender (men/women)	51/57	
Duration of hypertension (years)	10 (3–19)	
BMI (kg/m^2^)	30.0 ± 4.7	29.6 ± 4.9*
SBP/DBP (mmHg)	129 ± 18/ 75 ± 12	124 ± 16*/ 72 ± 10*
Fasting glucose (mg/dL)	111 ± 24	104 ± 27*
Hba1c (%)	6.1 ± 0.7	6.1 ± 0.7
Total cholesterol (mg/dL)	184 ± 35	176 ± 37*
HDL-c (mg/dL)	56 ± 15	54 ± 15*
LDL-c (mg/dL)	103 ± 30	96 ± 31*
Triglycerides (mg/dL)	115 (91–156)	121 (86–162)*
Creatinine clearance (mL/min)	106 ± 33	120 ± 46*
hs-CRP (mg/dL)	0.37 (0.24–0.65)	0.32 (0.15–0.53)*
ACE inhibitors/ARAII, n (%)	94 (87.0)	107 (99.1)*
Diuretics, n (%)	62 (57.4)	84 (77.8)*
Calcium antagonists, n (%)	68 (63.0)	87 (80.6)*
Statins/fibrates, n (%)	77 (71.3)	100 (92.6)*
Oral Anti-diabetic Agent /insulin, n (%)	33(30.5)	33(30.5)
Aspirin, n (%)	19 (17.6)	32 (29.6)*

Data are media ± SD or median (interquartile range). Abbreviations: BMI, body mass index; SBP, systolic blood pressure; DBP, diastolic blood pressure; CRP, C-reactive protein, ACE, angiotensin converting enzyme; ARAII, angiotensin II receptor antagonist

*P<0.05vsbaseline.

**Fig 1:**
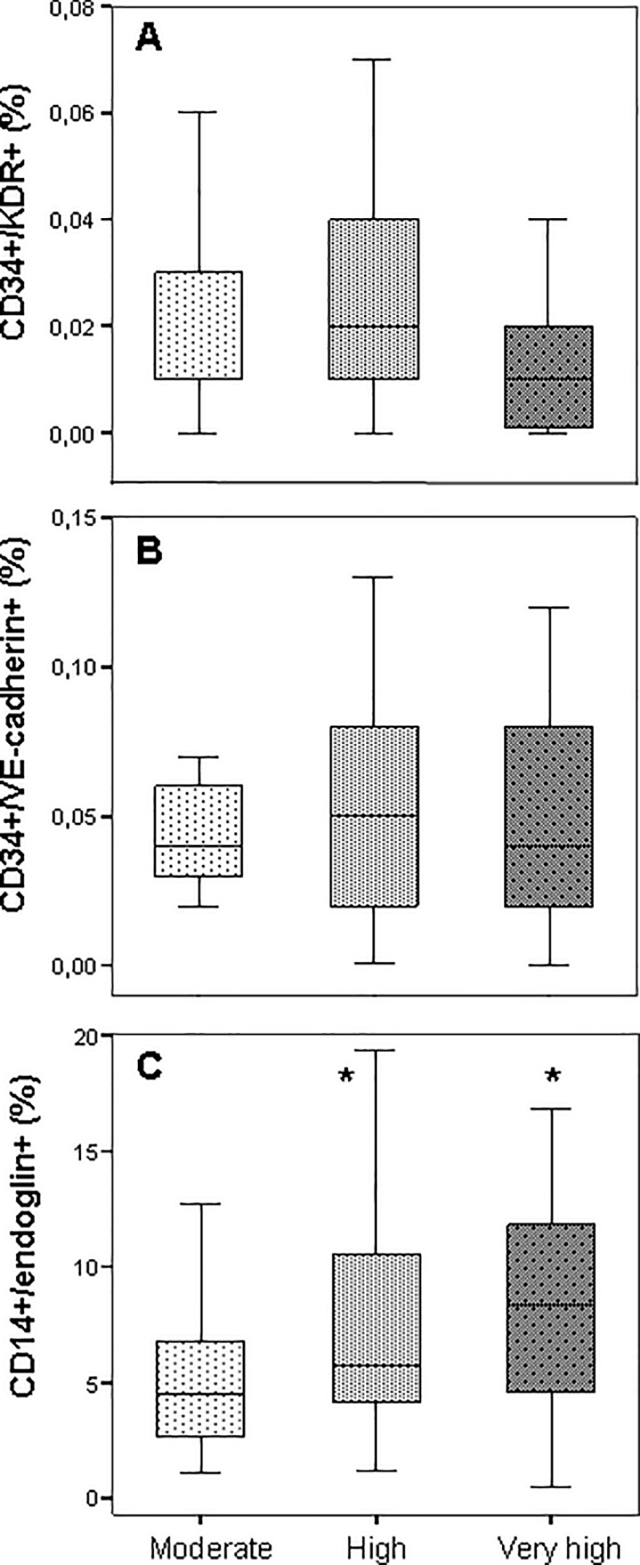
Circulating vascular progenitor cell levels in hypertensive patients according to cardiovascular risk stratification prior to intensive treatment. We divided the patients into three groups according to 2007 European Society of Hypertension/European Society of Cardiology (ESH/ESC) Guidelines for the management of arterial hypertension [26]: very high-risk patient were those having an established cardiovascular or renal disease, high cardiovascular risk when the patient had 3 or more risk factors, organ damage or diabetes mellitus, and moderate cardiovascular risk when the patient had one or two risk factors. Results are expressed as percentage of CD34+/KDR+, CD34+/VE-cadherin+ or CD14+/endoglin+ cells in the PBMC gated area once excluded CD3+ cells and data visualized as standard box plots. * P<0.05 vs moderate cardiovascular risk.

Regarding the cardiovascular risk stratification, CD34+/KDR+ and CD34+/VE-cadherin+ cell levels did not differ significantly when cardiovascular risk increases according to the model of qualitative cardiovascular risk stratification taken from 2007 ESH/ESC Guidelines (Fig 1A and 1B). However, there was a marked increase in CD14+/endoglin+ cells when the cardiovascular risk worsens (Fig 1C).

Table 2 describes the association between demographic characteristics, cardiovascular risk factors, and clinical and biochemical parameters of patients with the levels of progenitor cells CD34+/KDR+, CD34+/VE-cadherin+ and CD14+/endoglin+. Patients with a higher number (above 25^th^ percentile) of CD34+/KDR+ and CD34+/VE-cadherin+ and lower of CD14+/endoglin+ cells (equal or below 75^th^ percentile) were younger, with less years since diagnosis of hypertension, and with lower BP. These associations were maintained in a multivariate analysis. No differences were found regarding other cardiovascular risk factors. Interesting, patients with previous cardiovascular disease (ischemic stroke, myocardial infarction, symptomatic peripheral artery disease, some revascularization procedure, chronic kidney disease, and/or advanced retinopathy) tended to have higher number of CD14+/endoglin+ cells than those without a cardiovascular event.

**Table 2:** Clinical-biochemical parameters of hypertensive patients regarding levels of CD34+/KDR+, CD34+/VE-cadherin+, CD14+/endoglin+cells.

	CD34+/KDR+		CD34+/VE-cadherin+		CD14+/endoglin+	
	Low levels (n = 50)	High levels (n = 42)	Low levels (n = 25)	High levels (n = 64)	Low levels (n = 64)	High levels (n = 21)
Age (years)	63±12	58±11	66±9	60 ± 12*	61±12	63±8
Male (%)	45.6	54.3	56.0	45.3	42.2	71.4
BMI (kg/m2)	29±4	30±4	29±4	30 ± 4	29±4	29±5
Time of hypertension (years)	11 (4–19)	8.5 (2–17)	11 (6–19)	10 (2–17)	10 (3–19)	13 (3–17)
SBP (mmHg)	133±17	124±15*	133±16	128±16	127±15	135±20*
DBP (mmHg)	76±11	72±11	77±12	73±11	73±10	74±10
Fasting glucose (mg/dL)	111±26	109±18	111±21	109±23	109±24	114±23
Hba1c (%)	6.1±0.8	6.0±0.6	6.0±0.4	6.0±0.8	6.8±0.8	6.1±0.6
Total cholesterol (mg/dL)	178±28	187±41	180±32	183±36	185±37	185±31
HDL-c (mg/dL)	56±14	52±12	54±13	54±13	55±14	56±12
LDL-c (mg/dL)	97±23	108±±36	103±±25	101±±32	103±±32	105±±32
Triglycerides (mg/dL)	113 (93–150)	125 (75–175)	103 (83–127)	129 (92–172)*	118 (86–174)	108 (91–150)
Creatinine clearance (mL/min)	107±±32	107±±32	95±±25	112±±34*	104±±34	111±±27
hs-CRP (mg/L)	0.36 (0.22–0.57)	0.37 (0.27–0.72)	0.41 (0.28–0.64)	0.35 (0.22–0.65)	0.37 (0.24–0.65)	0.32 (0.21–0.46)
Previous cardiovascular disease (%)	38.0	26.2	36.0	31.3	26.6	47.6

Data are media ±± SD or median (interquartile range). Previous cardiovascular disease: Ischemic stroke, myocardial infarction, symptomatic peripheral artery disease and/or any revascularization procedure, chronic kidney disease, advanced retinopathy. Abbreviations: BMI, body mass index; SBP, systolic blood pressure; DBP, diastolic blood pressure; CRP, C-reactive protein.

* P< 0.05 vs low levels of the same progenitor subtype cells.

**Fig 2:**
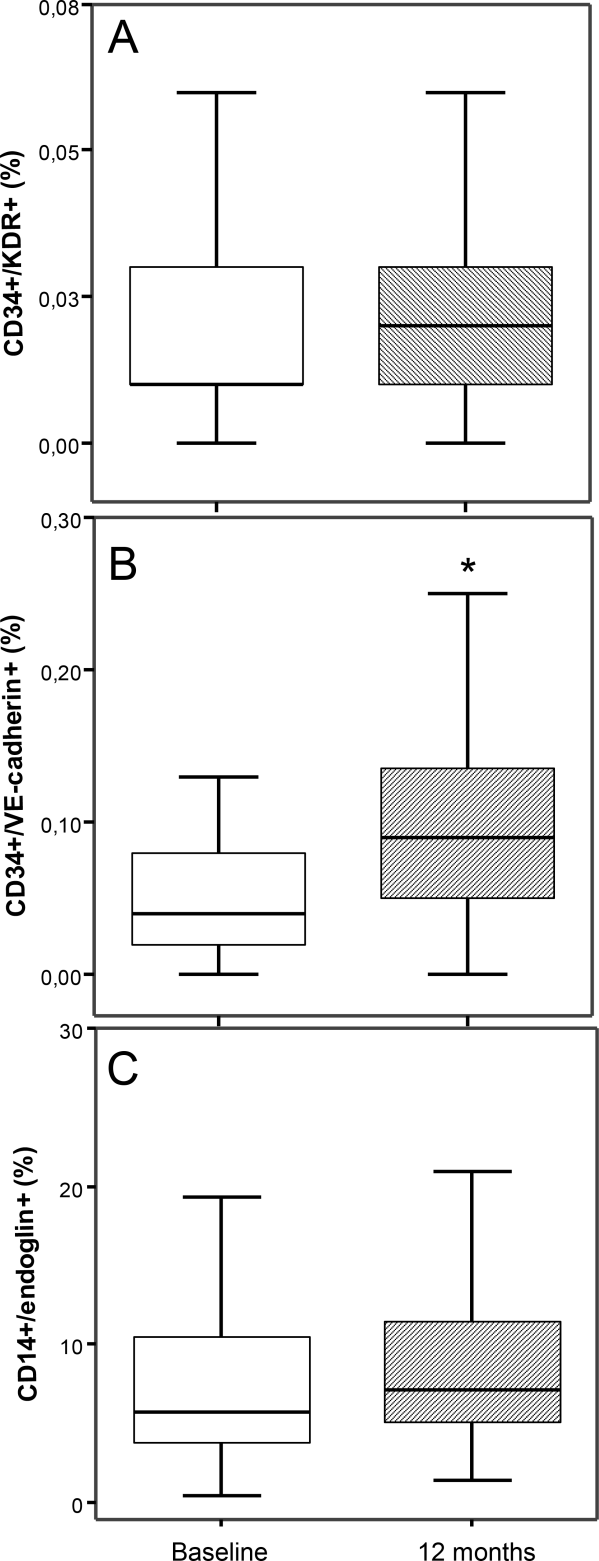
Circulating vascular progenitor cell levels in hypertensive patients before (baseline) and after treatment intensification during 12 months. Results are expressed as percentage of CD34+/KDR+, CD34+/VE-cadherin+ or CD14+/endoglin+ cells in the PBMC gated area once excluded CD3+ cells and data visualized as standard box plots, *P<0.05 vs before intensification of treatment.

The multifactorial intensive treatment for 12 months did not modify CD34+/KDR+ and CD14+/endoglin+ cells with respect to baseline (Fig 2A and 2C). By contrast, CD34+/VE-cadherin+ cells changed significantly after the treatment, increasing around 2-fold with respect to basal levels (Fig 2B).

### VEGF levels in hypertensive patients

**Fig 3:**
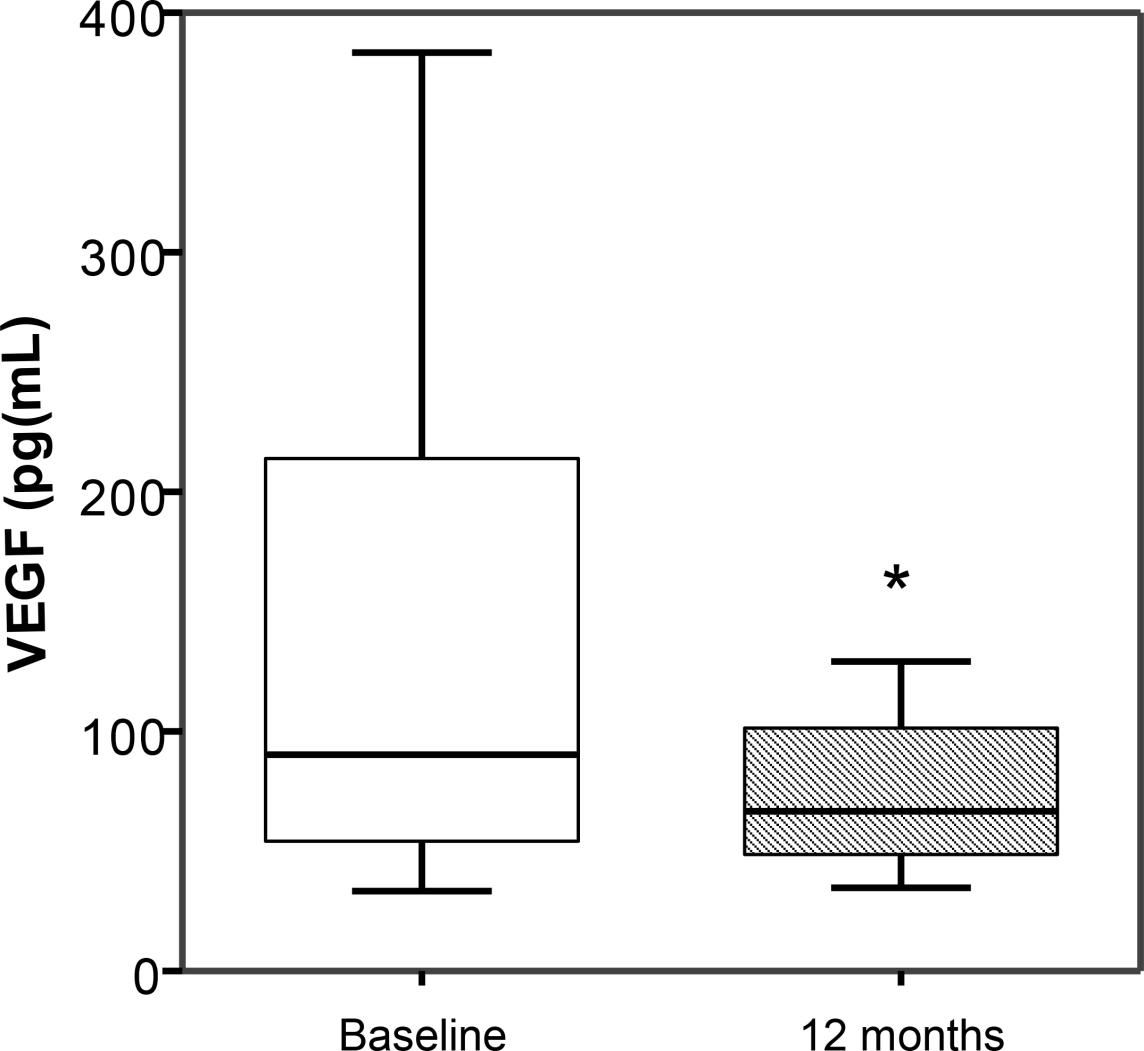
Plasma levels of VEGF in hypertensive patients before (baseline) and after treatment intensification during 12 months. Results are expressed as (pg/mL) of VEGF plasma levels. Data are visualized as standard box plots. * P<0.05 vs before intensification of treatment.

Since VEGF is one of the best characterized growth factors responsible of the mobilization of EPCs from the bone marrow [27], we investigated its plasma levels before and after the intensive treatment. As can be seen in Fig 3, VEGF plasma levels decreased after 12 months of intensive treatment.

## Discussion

Our data demonstrate that a long intensive multifactorial treatment aiming to achieve the highest therapeutic targets for traditional cardiovascular risk factors increases levels of circulating EPCs of hypertensive patients.

EPCs reduction or dysfunction has been inversely associated with traditional cardiovascular risk factors and with the development of future cardiovascular events, having been revealed as a new cardiovascular biomarker [12, 13]. Therefore, we focus this work on the analysis of the EPCs number since it has been considered a promising therapy in the treatment of cardiovascular diseases. It is well accepted that CD34+/KDR+ cells are less mature or early circulating EPCs, whereas more mature circulating EPCs are positive for CD34/VE-cadherin. At present, CD34+/KDR+ cells are the only EPC subtype that has demonstrated to have a strong association with cardiovascular risk [28].

Several studies have investigated the effect of drugs commonly used in the treatment of cardiovascular diseases on the number of EPCs and their functionality. Most studies have demonstrated that antihypertensive drugs such as ACE inhibitors, ARAII and calcium channel antagonists can improve the number and functionality of EPCs and that these effects are independent of the BP lowering effect [29–31]. Similarly, anti-diabetic drugs and statins, such as dipeptidyl peptidase 4 (DPP4) inhibitors and rosuvastatin respectively, may directly affects EPCs [32, 33]. Therefore, it could be speculate that the beneficial effects of these drugs in the outcome of cardiovascular patients should be due, at least in part, to their actions on EPCs.

In this study, we have included hypertensive patients with added cardiovascular risk that have received an intensive and multifactorial treatment. Hypertensive subjects often have a cluster of risk factors that greatly augments the cardiovascular hazard of elevated BP [2]. Therefore, the goal of therapy should be to improve the global risk profile of the patients and recent guidelines have factored into more aggressive therapeutic decisions [20]. Compared with optimal BP, high-normal BP is associated with 1.6–2.5 fold increased risk of presenting a cardiovascular event [26]. Our data demonstrate that intensive treatment aiming to achieve the highest therapeutic targets increased circulating CD34+/VE-cadherin+ cells. Although we did not find changes in CD34+/KDR+ and CD14+/endoglin+ cell levels, our data suggest a beneficial effect of the treatment on EPC mobilization. As we have commented before, many drugs used for managing of cardiovascular risk factors of our patients have been reported to increase the number and/or the functionality of EPCs, although most of these trials have evaluated short period of treatments (< 6 months) [20]. However, Schmidt-Luckeet al. reported that long-term administration of a statin predicts reduced numbers of EPCs in patients with CAD [34]. Furthermore, Deschaseaux et al. demonstrated that long-term statin therapy raises EPC levels by increasing EPC populations positive to CD34/VE-cadherin without affecting levels of cells characterized by expressing CD34/KDR [35]. These results suggest that the effects of statins on EPC mobilization may be transient. In early stages, statins help mobilize EPCs CD34+/KDR+, which is especially important in conditions of acute ischemia, but in the long-term, they increase CD34+/VE-cadherin+ cells, considered as the "true" EPCs responsible for vasculogenesis [35], and providing a mechanism for the beneficial effects of these drugs. Unfortunately, we did not measured EPCs at short times, although this is a very interesting aspect that deserves further studies.

The mechanisms by which the intensification of drug treatment of hypertensive patients may have a beneficial effect on circulating EPCs are not well-known. Cardiovascular risk factors cause oxidative stress that alters VEGF regulation by preventing the attachment to its receptor on bone marrow [36]. Our patients showed elevated plasma levels of VEGF associated to decrease levels of EPCs, meanwhile intensive treatment-induced circulating EPCs mobilization was associated to a significant reduction of plasma VEGF concentration, suggesting that a further reduction of oxidative stress could be occurring. In this sense, several drugs, including ACE inhibitors and statins, have demonstrated antioxidant and anti-inflammatory actions and a protective effect against endothelial dysfunction through different mechanisms, and their combination achieves additional beneficial effects than monotherapies [37–39]. However, we cannot rule out that the combination of drugs and/or increasing doses might have a stimulatory direct effect on bone marrow.

In this paper we have also shown that hypertensive patients had an increase in the SMPCs CD14+/endoglin+ that did not change after the intensive treatment. Although several studies have shown the presence of SMPCs in atherosclerotic lesions [24, 40], their exact role is not too clear. There is some evidence showing that these cells can contribute to the development of vascular disease. Patients with previous cardiovascular disease had higher numbers of CD14+/endoglin+ cells than those without previous cardiovascular disease [24]. However, in several experimental models, the administration of SMPCs limited the development of atherosclerotic lesions and induced plaque stabilization [41, 42]. In patients with acute coronary syndrome, SMPCs deficiency has been associated with plaque vulnerability [22]. A previous study from our laboratory has shown that in HIV patients, the profile of CD34+/KDR+ and CD34+/VE-cadherin+ diminished and CD14+/endoglin+ increased is associated with higher cardiovascular risk [43]. However, longer longitudinal studies are required to establish what might be the role of CD14+/endoglin+ cells in the process of atherosclerosis in patients with hypertension.

Our study has some limitations. We have not evaluated EPC functions in this study since our aim was to investigate the variation in the number of circulating vascular progenitor cells after 12 months of intensive therapy in order to justify its use as a biomarker of therapeutic efficacy, requiring to this purpose simple, rapid and reproducible methods as flow cytometry. During our study, only one patient suffered a cardiovascular event. These patients are currently being followed up in our Unit of Hypertension in order to evaluate whether changes in the number of EPCs showed in this study translate into a reduction of cardiovascular events. In addition, we know that in multifactorial interventions are not possible to clear out which part has been most efficient. Although, previous studies have indicated that most of the CVD benefit came from statin therapies[44].

In conclusion, we have demonstrated that EPCs can be good therapeutic target in hypertensive patients, suggesting a new potential pharmacological mobilization therapy. In this sense, the use of cellular therapeutic strategies has emerged as a powerful tool contributing to cardiovascular regeneration. Thereby, different routes of EPCs administration such as autologous EPC injection, pharmacological mobilization and EPCs capture stents have demonstrated to stimulate the revascularization of ischemic vessels in different animal models of limb ischemia or myocardial infarction However the clinical transfer of EPC-based therapies has shown conflicting results, with only some trials reporting an improvement of cardiac function and a reduction of important clinical end-points [45, 46]. Many reasons could account for these data, including different patient characteristics and EPCs sources and/or phenotypes. In this sense, there are not specific cell surface markers that precisely identify the different subtypes of EPCs, resulting in the use of different EPCs populations to induce regeneration. In addition, the efficacy of bone marrow-derived EPCs over peripheral blood-derived EPCs (or vice versa) is still lacking. On the other hand, EPCs from patients with high cardiovascular risk are known to be numerical and functionally impaired. It is possible that in the future, treatment of these patients will require therapy aimed to restore directly the progenitor cell reservoir in the bone marrow, although its regenerative efficacy needs to be elucidated.
